# Improving women's nutrition imperative for rapid reduction of childhood stunting in South Asia: coupling of nutrition specific interventions with nutrition sensitive measures essential

**DOI:** 10.1111/mcn.12255

**Published:** 2016-05-17

**Authors:** Sheila C. Vir

**Affiliations:** ^1^ Public Health Nutrition and Development Centre New Delhi India

**Keywords:** women's nutrition and anthropometry, low birth weight, IUGR, stunting, women's empowerment, nutrition specific, nutrition‐influencing factors

## Abstract

The implications of direct nutrition interventions on women's nutrition, birth outcome and stunting rates in children in South Asia are indisputable and well documented. In the last decade, a number of studies present evidence of the role of non‐nutritional factors impacting on women's nutrition, birth outcome, caring practices and nutritional status of children. The implications of various dimensions of women's empowerment and gender inequality on child stunting is being increasingly recognised. Evidence reveals the crucial role of early age of marriage and conception, poor secondary education, domestic violence, inadequate decision‐making power, poor control over resources, strenuous agriculture activities, and increasing employment of women and of interventions such as cash transfer scheme and microfinance programme on undernutrition in children. Analysis of the nutrition situation of women and children in South Asia and programme findings emphasise the significance of reaching women during adolescence, pre‐conception and pregnancy stage. Ensuring women enter pregnancy with adequate height and weight and free from being anemic is crucial. Combining nutrition‐specific interventions with measures for empowerment of women is essential. Improvement in dietary intake and health services of women, prevention of early age marriage and conception, completion of secondary education, enhancement in purchasing power of women, reduction of work drudgery and elimination of domestic violence deserve special attention. A range of programme platforms dealing with health, education and empowerment of women could be strategically used for effectively reaching women prior to and during pregnancy to accelerate reduction in stunting rates in children in South Asia.

**Key messages**

- Towards reducing stunting in South Asia, programme efforts need to ensure that women enter pregnancy with optimum height, adequate weight and free from anemia. (24)
- Combining improving coverage of specific nutrition interventions for women and children with intensification of nutrition sensitive measures for women such as preventing early marriage and conception, promoting completion of secondary education, improving socio‐economic status and control over resources, improving access to water, sanitation and cooking fuel facilities and reducing physical workload is essential. (53)
- The implications of dual burden of overweight and anemia in women living in poor economic environments of South Asia on childhood stunting needs to be explored. (25)

Maternal undernutrition is estimated to account for 20% of childhood stunting (WHO 2014). Women's nutrition plays a crucial role in optimising pregnancy outcome and influencing maternal, neonatal and child health outcomes (Mason *et al.* 2012). Low status of women in South Asia has been postulated to be a significant contributor to the unusually high rate of undernutrition in children in South Asia (Ramalingaswamy *et al.* 1996). Poor socio‐economic status of women not only affect fetal growth and pregnancy outcome but also adversely impacts behavioural practices pertaining to appropriate self and child care, which contribute to low body mass index (BMI) in women and stunting in children. Today, there is increasing evidence and recognition among the scientific community that it will be difficult to achieve rapid and significant progress in reducing childhood stunting without scaling up evidence‐based direct nutrition interventions as well as simultaneously addressing the underlying socio‐economic causes that adversely influence nutrition of women (Bhutta *et al.* 2008; Ramakrishnan *et al.* 2012; Smith & Haddad 2015). Women's nutrition, a low priority in the public health agenda of most developing countries, including South Asia, needs special attention for accelerating reduction of stunting rates in children (Ramakrishnan *et al.* 2012; Saldana *et al.* 2012). This review paper describes the current nutritional status of women in South Asia, linkage of women's anthropometry with birth outcome and stunting in children, and presents evidence of the nutrition‐specific and nutrition‐sensitive interventions including varied dimensions of empowerment of women, which collectively play a crucial powerful role in high rates of undernutrition in South Asia.

## Women's anthropometry, intrauterine growth restriction, low birth weight and stunting

**Figure 1:**
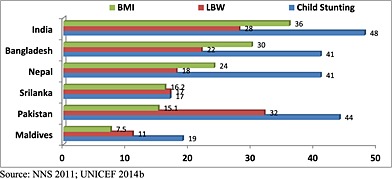
Percentage of women 15–49 years old with low body mass index (BMI), incidence of low birth weight (LBW) and percentage of stunted children under 5 years old in South Asian countries.

Women's poor nutrition, both before and during pregnancy, contributes to impairment of fetal development and contributes to low birth weights (LBW) and in turn to high rates of stunting. A global analysis of region‐wise data between the 1980s and 2000s, including South Asia, reveals that improvement in BMI of women 15–49 years corresponds with a reduction in the rate of LBW (Mason *et al.* 2012). In South Asia, the percentage of women with low BMI indicating undernutrition ranges from 7.5% in Maldives to over a third of women (36%) in India (Fig. 1). Except in the case of Pakistan and Sri Lanka, the other South Asia countries are noted to have a high percentage of women with low BMI and a corresponding high percentage rate of LBW and stunted children (NNS 2011; UNICEF 2014b). Interestingly in the case of Pakistan, Sri Lanka and Maldives, the prevalence rate of overweight and obesity in women in the reproductive age is high, and despite such a situation, the incidence of LBW is over 10%. Overweight women living in poor economic environment are often suffering from anaemia with possible adverse impact of the dual burden on fetal growth. This needs to be further explored. Improvement in the maternal nutrition situation in Nepal and Bangladesh, on the other hand, is reported to result in larger birth size with substantial contribution in reducing undernutrition in children (Headey & Hoddinott 2014; Headey *et al.* 2014).

In the last decade, an association of maternal anthropometry (height, weight or thinness) and birth weight has been stressed (Black *et al.* 2013). Maternal stunting (height < 145 cm) increases the risk of both term and preterm small for gestational age (SGA) babies (Black *et al.* 2013). Pooled analysis of 7630 mother–child pairs from birth cohorts of five countries, Brazil, Guatemala, India, Philippines and South Africa, reveals that maternal height is associated with birth weight and with linear growth over the growing period. Short mothers (<150 cm) are reported to be three times more likely to have a child who is stunted at 2 years of age and as an adult (Addo *et al.* 2013). An analysis of national demographic survey findings from India reveal a significant decrease in relative risk of stunting in children for every 5 cm increase in maternal height from <145 to >160 cm (Subramanian *et al.* 2009). Similar findings have been reported from an analysis of 109 demographic surveys undertaken between 1991 and 2008 in 54 countries with a large sample comprising 2 661 519 children born to 751 912 mothers (Ozaltin *et al.* 2010). This study also reports that the effect size of short maternal height is twice that of being in the lowest education category and 1.5 times that of being in the poorest quintile. The significance of women being provided appropriate and timely inputs for attaining optimum adult height is evident (Subramanian *et al.* 2009).

Besides poor height or stunting in mothers, significance of weight of women on birthweight is important. In India, mean birth weights of infants born to mothers below 45 kg is reported to be about 2.7 kg as compared with mean birth weight of 2.9 kg in mothers weighing 45–54 kg compared with 3.1 kg in case of mothers 55 kg and above (Ramachandran 1989). Based on a meta‐analysis of maternal anthropometry, pre‐pregnancy weight is considered a good predictor of LBW and a pre‐pregnancy weight of less than 40 kg is proposed as a useful cut‐off to predict women who have a high chance to deliver LBW babies (Tontisirin & Bhattacharjee 1999). A recent prospective study from Vietnam concludes maternal pre‐pregnancy weight to be the strongest indicator predicting infant birth size (Young *et al.* 2015). Women with pre‐pregnancy weight less than 43 kg or who gained <8 kg during pregnancy are reported to be more likely to give birth to an SGA or LBW infant. Well‐designed prospective cohort studies in other developing country settings need to be undertaken to systematically examine the relationship between pre‐pregnancy body size and composition and maternal nutrition and child health outcomes (Ramakrishnan *et al.* 2012).

South Asia carries 52% of the global burden of LBW (UNICEF 2013). Globally, three of the five countries with an incidence of LBW of over 20% are from South Asia – Pakistan, India and Bangladesh (Fig. 1). The situation of LBW in South Asia is possibly much worse because timely and accurate weighing of newborns is a low public health priority and far from a universal practice. In India, only three‐fourths of the newborns are reported to be weighed before discharge (JSY 2011; UNICEF 2013), and birth weight is often recorded to a rounded figure of 2500 g to avoid any queries or follow‐up management efforts required. Moreover, reporting of LBW incidence does not present the true dimension of the problem of implications of poor nutrition of women on birth outcomes. LBW measure underestimates the problem of fetal growth restriction or intrauterine growth restriction (IUGR). The SGA measure is considered more appropriate for assessment of problem of poor birth outcome. For instance, using this measure, 46.9% of births in India are estimated to be SGA as against 28% reported LBW (Black 2013, NFHS 3).

Low birth weight children often do not recover from poor start in life and contribute to high rate of stunting in early childhood (Sachdev 2011). An analysis of data of low‐income and middle‐income countries indicate LBW is associated with 2.5‐fold to 3.5‐fold higher odds of wasting, stunting and underweight in children (Christian *et al.* 2013). Impaired fetal development increases the risk of stunting 2.1 to 4.3 times (Sachdev 2011). It is estimated that newborns who are SGA and term have an odds ratio of 2.43 for stunting at 12–60 months, while being SGA and preterm increases the odds ratio to 4.5% (Christian *et al.* 2013). IUGR infants generally fail to catch up to normal size during childhood (Martorell *et al.* 1998). Stunting attributable to LBW is highest in the first 6 months – the risk of stunting decreases with increase in age. It is estimated that with 30% LBW in India, child stunting rate attributable to LBW is 37% at 6 months and 13–22% at age 1–5 years (Sachdev 2011). There is adequate evidence supporting the fact that stunting begins in utero, and newborn size is a strong predictor of achievement of height at 12 months (WHO 2014).

## Life cycle of women: critical periods impacting childhood stunting

The major increase in the rate of stunting in South Asia, as in other developing worlds, takes place during the period of gestation to approximately 24 months post‐delivery (Black *et al.* 2008). Growth failure in the first 1000 days of life (conception to 2 years) is a strong determinant of adult height (Victora *et al.* 2010). The prevalence rate of stunting increases rapidly in the first 2 years of life and reaches its peak at about 2 years of age, and the poor growth during this period is largely irreversible. The age‐wise data from India reveal 57.8% children are stunted at 18–23 months compared with 20.4% at 6 months 32.0% at 9–11 months and 46.9% at 12–17 months (NFHS‐3 2006). A similar pattern of growth and rates of stunting increasing in the first 2 years of life is reported from Pakistan and Maldives (DHS‐Maldives 2009; NNS 2011). Recent analysis of nationally representative data of Bhutan reports children 12–23 months have a threefold odds of being stunted compared with infants 0–11 months (Aguayo *et al.* 2015).

**Figure 2:**
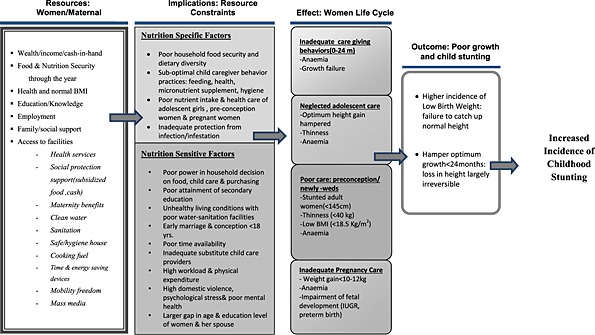
Constraint on women's resources:: implications on nutrition‐specific and nutrition‐sensitive factors and childhood stunting.

Stunting that occurs in children under 2 years old is largely irreversible. Female children who are stunted in early age therefore have a higher chance of growing up to be stunted adult women. This sets up an intergenerational cycle of undernutrition in women. Care of children 0–24 months is essential to prevent linear growth retardation in early childhood in low‐income and middle‐income countries (Shrimpton *et al.* 2001). Recent findings reported by WHO provide evidence that short‐term improvement in nutrition, extending from intrauterine life to the first 24 months of childhood, can in fact result in mean gain in adult height of 8 cm greater than mean parental height in just one generation in low‐income and middle‐income countries (Garza *et al.* 2013). This finding is encouraging evidence that a faster trans‐generational improvement in height is achievable in just one generation than has been assumed earlier (WHO 2014). Appropriate infant and young child feeding, prevention of infection, childhood stimulation in the first 2 years of life is crucial. These child care practices are influenced to a great extent by maternal care resources (Fig. 2), which are not limited to mother's nutrition and physical well‐being but to factors that influence mother's empowerment such as education and knowledge, decision‐making power and control over household resources, employment and time availability (UNICEF 1990; Engle *et al.* 1997). In South Asia, a recent review study indicates that the following three domains of women empowerment, i.e. control of resources and autonomy, workload and time, and social support environment, influence child anthropometry, but the strength and direction of association is reported to differ in the contextual situation including child's age and household wealth (Cunningham *et al.* 2015).

The other most important periods in the life cycle that are critical and influence the rate of LBW and in turn childhood stunting are inadequate adolescence care, neglected pre‐conception care and poor care and weight gain during pregnancy (Fig. 2). Adolescence is the period of second and last growth spurt, and the final height in adulthood is influenced by gain in height during this period. Optimum gain in height during adolescence in girls is adversely influenced by the onset of conception at young age. The adverse impact of early conception on optimum growth is much worse in a disadvantaged population where the velocity of adolescent growth is slower and is extended for a longer period (Vir 1990). As described later in the paper, adolescence conception as well as inadequate diet and health care hamper optimum height gain resulting in adolescent girls entering adulthood with short stature, poor weight and anaemia with its adverse impact on fetal growth resulting in LBW and stunting. This is further supported by a recent report of a prospective study on data pooled from five low middle‐income countries, including India, which demonstrated a stronger association of younger maternal age with lower birth weight, preterm birth and stunting by 2 years of age as compared with such an association in the case of women 20–24 years (Fall *et al.* 2015).

Besides neglected care in the adolescence stage of life, poor pre‐pregnancy care or pre‐conception care resulting in poor weight also contribute to LBW and stunting (Fig. 2). In South Asia, women often enter pregnancy not only with inadequate height but often with low weight with serious adverse impact on optimum growth of fetus resulting in high incidence of LBW and contributing to high incidence of stunting. A WHO collaborative study on maternal anthropometry and pregnancy outcomes reports that mothers in the lowest quartile of pre‐pregnancy weight carried an elevated risk of 2.55 for IUGR and 2.38 for LBW compared with the upper quartile (WHO 1995). This study also showed that attainment of inadequate maternal weight in 20, 28 and 36 weeks of gestation also raised the risk of IUGR. The findings provide evidence that women in the lowest quartile for both pre‐pregnancy weight and weight gain during pregnancy are at the highest risk of producing IUGR infants. This is confirmed by the recent large prospective study from Vietnam, which reports high risk of delivering SGA or LBWs among women who are underweight and with low BMI in pre‐conception stage (Ramakrishnan *et al.* 2012; Young *et al.* 2015). The study also emphasises the significance of adequate weight gain during pregnancy. Addressing women's weight prior to onset of pregnancy is crucial and cannot be ignored.

## Undernutrition in women and stunting in children: nutrition‐specific and nutrition‐sensitive issues

Women influence their children's nutritional status through their impact on pregnancy outcomes as well as through effect on child care practices (Smith *et al.* 2003 & Bold *et al.* 2013). Poor dietary intake and poor availability of nutrients consumed due to ill health are well‐known immediate and direct causes of undernutrition in women with serious contribution to undernutrition in children. These immediate causes are influenced by a number of underlying socio‐economic factors such as purchasing power, gender inequality, decision‐making power of women at family level, and investment in nutrition care of self, children and family (Fig. 2). In the last decade, there is increased evidence of such factors influencing women's nutrition and its association with nutritional status of children. The available evidence, described subsequently, underlies the significance and the need to simultaneously address both direct nutrition‐specific and nutrition‐influencing factors.

## Direct nutrition‐specific factors, women's nutrition and childhood stunting

### Energy imbalance, poor food diversity and stunting

Dietary intake of women in South Asia is observed to lack energy and diversity not only during pregnancy but also prior to onset of pregnancy. Rural India data reveal that consumption of mean energy and protein is almost identical in pregnant (1773 cal and 49 g protein) and adult non‐pregnant women (1709 cal and 47 g). Only 61% of pregnant women report consuming over 70% of the recommended dietary allowances (RDA) of energy, while only 30% consume over 70% RDA of protein. No increase in intake of iron, vitamin A and calcium is observed during pregnancy with less than 10% consuming >70% RDA of iron and calcium, while only 13% are reported to be consuming >70% RDA of vitamin A (NNMB 2012).

Poor diet diversity during pregnancy has been identified as an important factor that needs to be addressed for reducing prevalence rate of stunting in South Asia (Smith & Haddad 2015). Lack of attention to increasing dietary intake during pregnancy could be attributed to poor purchasing power, inadequate information on the significance of additional requirements of energy and various nutrients during pregnancy as well as an incorrect common cultural practice of ‘eating down’ during pregnancy, which is prevalent in some regions of India and possibly in the neighbouring South Asia countries. Primary reason for poor dietary intake seems to be lack of knowledge regarding appropriate dietary care during pregnancy. This is evident from the fact that intensive counselling to pregnant mothers in Northern India resulted in significant increase in calorie consumption (Garg & Kashyap 2006). A recent randomised control trial from Bangladesh also reports that monthly education sessions, promoting consumption of local food item ‘*Khichuri*’ during the third trimester of pregnancy, resulted in maternal weight gain in the third trimester to be 60% higher, mean birth weight 20% higher and the rate of LBW to be 94% lower in the intervention group compared with control (Khurshid *et al.* 2014). A recent report from Southern India of a large‐scale innovative trial of providing one hot cooked meal per day with diversified food items at a subsidised rate to pregnant women along with nutrition education resulted in a much higher increase in weight gain during pregnancy and reduction in the incidence of LBW (Chava 2012). Meta‐analysis reports a significant reduction of 31% in the risk of giving birth to SGA infants when pregnant women are provided with balanced protein energy supplements (Imdad & Bhutta 2011). Targeting of mothers having low BMI with supplement of more than 700 kcals per day is estimated to reduce SGA by 32% (Bhutta *et al.* 2008). Dietary supplement providing 25% of energy as protein is crucial and is reported to increase birth weight by 73 g and reduce SGA by 34% (Black 2013). On the other hand, questions have also been raised regarding the functional consequences of such maternal supplement to thin women (Kramer 2003).

Besides dietary intake, excessive energy expenditure due to heavy workload adversely influences pre‐pregnancy weight, BMI of women and gestational weight gain during pregnancy. Studies have demonstrated that in situations where energy intake is suboptimal, manual physical activity during pregnancy lowers weight gain during pregnancy with increase in incidence of SGA and lower birth weight babies (Tafari *et al.* 1980; Launer *et al.* 1990). In rural India, high levels of daily physical activity, related to agriculture and domestic tasks, have been reported to have an inverse relationship with birth weight (Rao *et al.* 2003). A direct relationship between maternal physical activity and birth weight has also been reported (Muthayya 2009). Working in farms or fetching water are other activities that are reported to have a significant inverse relationship to birth size even after adjusting for maternal co‐founding factors (Rao *et al.* 2003). Farming activities reveal a seasonal energy stress on women depending on lean or harvesting agriculture period with its impact on energy balance and impact on pregnancy outcome. Reduction in activity during harvest season, when food is in plenty, has been proposed for improving birth size of farming communities.

The other emerging problem in South Asia is increase in rate of overweight and obesity in women. In Maldives and Sri Lanka, over a third of women are overweight or obese (UNICEF 2014b). A high rate of overweight is also noted in four states (Kerala, Goa, Punjab and Delhi) of India with over a quarter of women reported being overweight (NFHS3). Recent analysis of nationally representative data from Pakistan reveals that in 106 of 143 districts, more women are overweight than underweight (Cesare *et al.* 2015). Implications of such a trend of increase in rate of overweight in women on birth outcome or stunting rates in children in South Asia have not been systematically explored and deserve attention.

### Micronutrient deficiencies, anaemia and stunting

Requirements for micronutrients increase substantially during pregnancy, and maternal micronutrient deficiencies of iron and iodine are reported to be associated with adverse birth outcome, including LBW (Ramakrishnan *et al.* 2012; Zimmerman 2012). Maternal iron deficiency anaemia prior to and early pregnancy places the mother at increased risk of significant decrements in fetal growth, preterm birth or LBW delivery (Allen 2000; Scholl 2005). In South Asia, most women enter pregnancy anaemic. Prevalence rate of anaemia among adolescent girls is over 40% in all South Asia countries, except Bhutan which has a comparatively lower prevalence rate of 26.4% (WHO SEARO 2011). Anaemia rates in non‐pregnant women is also reported to be high in most of the large South Asia countries – 25% Afghanistan, 46% Bangladesh, 55% India, 36% in Nepal, 28% Pakistan and 16% in Maldives (UNICEF 2007; UNICEF 2009). The anaemia situation worsens during pregnancy with higher requirements for iron. It is estimated that on average, 56% of pregnant women in developing countries are anaemic compared with 18% of pregnant women in developed countries (Allen 2000; Abu‐Saad & Fraser 2010).

The primary reason for the high prevalence rate of anaemia is poor intake of dietary iron, low availability of iron from cereal‐based diet and poor consumption of animal foods or haem iron due to cultural practices or cost in most South Asian countries (WHO 2011). Data from Pakistan indicate higher intake of iron compared with the RDA, but the source of iron is primarily from wheat, which is not biologically available (NNS 2011). In rural India, only 23.0% adolescent girls, 15.2% adult women and 9.6% pregnant women are reported to consume over 70% RDA of iron (NNMB 2012). The main source of iron in India and other South Asia countries is cereals. In Maldives, despite regular but low consumption of animal source food, anaemia remains a public health problem but of much less magnitude than other South Asian countries (UNICEF 2007; DHS‐Maldives 2009).

It is also well established that deficiency of iron in the first trimester of pregnancy results in significant decrements in fetal growth and is generally more damaging to pregnancy outcome than iron deficiency anaemia in the second or third trimesters (Abu‐Saad & Fraser 2010). Iron supplementation is documented to have a significant effect on LBW (Balarajan *et al.* 2013; Khanal *et al.* 2014). In Nepal, mothers not consuming iron supplement during their pregnancy are reported to more likely have LBW babies (Imdad & Bhutta 2012). In each of the eight South Asia countries, provision of daily iron‐folic acid (IFA) supplements to pregnant women is an integral part of antenatal care (ANC) services. Coverage and compliance of IFA is low with only 44% of pregnant women in South Asia reported using IFA supplements compared with 53% globally (Gwatkin *et al.* 2007; UNICEF 2014b). Deworming in the second trimester of pregnancy in Nepal has been reported to lower the rate of severe anaemia and improve birth weight (Christian *et al.* 2004).

The significance of women entering pregnancy with adequate iron nutrition is well recognised, and weekly IFA supplements (WIFS) for prevention of anaemia in adolescent girls and women in reproductive age group are recommended (WHO 2009). The WIFS policy is already in place in India, Bangladesh, Sri Lanka and Bhutan (UNICEF 2014a). Successful lessons synthesised from global WIFS experience have been incorporated in the operational guidelines of these countries (WHO 2012). The benefits of WIFS are not limited to improvement in outcomes of pregnancy but have implications on improving concentration at work, school retention and education (WHO 2014).

Multiple micronutrient supplementation (MMNS) has been reported to reduce LBW by about 10% in low‐income countries (Fall *et al.* 2009). A hospital‐based trial from India in pregnant women enrolled at 24–32 weeks of gestation with low BMI and anaemia reports positive impact of adding on MMNS to the regular IFA supplement on improving birth weight by 98 g and increasing birth length by 0.80 cm and a substantial decline in LBW as compared with the placebo group (Gupta *et al.* 2007). Such positive impact of MMNS has also been reported from Nepal (Viadya *et al.* 2008), while Bangladesh reports benefits only when mothers have low BMI (Tofail *et al.* 2008). Replacing IFA with MMNS during pregnancy requires undertaking large‐scale effectiveness trials in the South Asia situation to rule out possible adverse impact on neonatal and perinatal mortality in disadvantaged population (Bhutta *et al.* 2012). There is also a need for developing a suitable MMNS product with composition suitable to meet the required gap in micronutrient intake in countries of the South Asia region.

The association of vitamin A deficiency with IUGR is not consistent (Lyman‐Thorne & Fawzi 2012). Reports on consumption of micronutrient‐rich foods such as green leafy vegetables and milk, even after adjusting for maternal co‐founding factors, are reported to have a significant association with birth weight (Rao *et al.* 2001). Pune Maternal Nutrition and Foetal Growth Study (PMNS) from India reports birth size is not associated with energy or protein intake but is associated with consumption foods rich in micronutrients. Another study from Northern India reports variation in mean birth weight of babies born during different seasons of the year and has demonstrated an association of incidence of birth weight with availability of seasonal fresh fruits and vegetables and consumption of micronutrients during pregnancy (Tamber 2006). Consumption of green leafy vegetables and locally available seasonal fruits appears crucial for improving micronutrient intakes and improving birth size even when energy intakes are limited during pregnancy. Deficit in non‐cereal food supply in South Asia diet with only 40% of the food supply being made up of non‐staples such as meats, fruits and vegetables is considered to be a primary contributor of poor women's nutrition and birth outcome (Smith & Haddad 2015). Extremely poor knowledge and negligible consumption of foods rich in micronutrients such as vegetables and fruits by women in reproductive age is reported from Maldives despite a significant improvement in economic and education situation (NMS 2007; DHS‐Maldives 2009). Reducing emphasis on cereals and improving dietary diversity of food in South Asia is recommended to be accorded a special focus for improving women's nutrition and reducing stunting rates in children (Smith & Haddad 2015).

With the adverse impact of iodine deficiency on fetal and post‐natal growth and development of young children, regular use of iodized salt is recommended (Zimmerman 2012). In the last two decades, iodised salt intake in six of the eight countries of South Asia has improved significantly – 69% in Pakistan, 71% in India, 80% in Nepal, 88% in Bangladesh, 92% in Sri Lanka and 96% in Bhutan. Intake of iodised salt is reported to be low in two South Asia countries – 20% in Afghanistan and 44% in Maldives (NNS 2011; UNICEF 2014b).

### Antenatal care services

Antenatal care services are likely to influence improvements in dietary practices, weight gain and introduction of timely interventions for preventing LBW (Hueston *et al.* 2003; Khanal *et al.* 2014). An analysis of demographic data of Nepal indicates that non‐attendance to ANC clinics increases the odds of having LBW by more than twice (Khanal *et al.* 2014). Studies in Bangladesh also report that odds of stunting are much higher in cases where mothers do not receive ANC or services at delivery are not provided by a skilled health professional (Hong *et al.* 2006). In Bhutan, 31% higher odds of being stunted is noted in case of children whose mothers received three or fewer ANC visits while children whose mothers received ANC other than from a trained professional are reported to have 51% higher odds of being stunted (Aguayo *et al.* 2015). Only 35% women in South Asia are reported to have at least four ANCs compared with almost 80% in East Asia and Pacific and 53% globally (UNICEF 2014b).

## Nutrition sensitive factors and stunting in children

Analysis of demographic surveys of three South Asia countries (Table 1) reveals that the highest‐risk factors influencing rate of stunting across these countries pertain primarily to a range of issues pertaining to women. These comprise women's health care, education, maternal height, domestic violence experience besides low standard of living, wealth quintiles and access to water (Adhikari *et al.* 2014; Headey & Hoddinott 2014; Headey *et al.* 2014). Maternal height, to a great extent, influenced by social issue of early marriage is a high‐risk factor for childhood stunting in all these countries (Table 1). These findings concur with the analysis of attributing factors of outstanding decline in child stunting in Brazil where social investments and public policies with universal education of women explain 25.7% of the decline in child undernutrition, while 21.7% of undernutrition reduction is attributed to substantial increase in purchasing power, 11.6% to maternal and child health care services and the remaining 43% to improvement in outreach of water and sewage facilities (Monteiro *et al.* 2007; Monteiro 2009). A recent report attributes the reduction in the prevalence rate of stunting in South Asia in the past four decades to substantial improvement in women's education as well as progress in the gender life expectancy ratio besides considerable increase in access to safe water (Smith & Haddad 2015).

**Table 1:** Highest‐risk factors associated with stunting in young children in India, Nepal and Bangladesh

Risk factors for stunting		
India	Bangladesh	Nepal
No education of mothers	Domestic violence	Maternal height
Maternal height	Decision‐making power	Water
Mothers with no institutional delivery	Maternal height	Open defecation
Households with low standard of living	Secondary education	Born in hospital
Households with no toilet facility	Wealth quintile	ANCs visits or more
—	—	Maternal education

Source: Headey & Hoddinott 2014; Headey *et al*. 2014 and Adhikari *et al*. 2014

### Decision‐making power of women, gender inequality and undernutrition in children

An analysis of 36 nationally representative data sets of demographic and health surveys of three developing regions (South Asia, sub‐Saharan Africa and Latin America and the Caribbean) confirms that women's decision‐making power relative to men has a powerful effect on nutritional status of children (Smith *et al.* 2003). The impact in South Asia is reported to be through the following two pathways that are influenced by empowerment and a higher decision‐making power – firstly through improvement in self‐care and prenatal care, and secondly through positive influence on behavioural and caring practices such as timely initiation of breastfeeding, complementary feeding (timely introduction and quality care), treatment of illness, immunisation and quality of substitute caretaking. The study concludes that if women and men had equal status in South Asia, with other factors remaining unchanged, the percentage of underweight children would be reduced by 13 percentage points (from 46 percent to 33 per cent) roughly 13.4 million children (Smith *et al.* 2003).

It has been reported that in situations where women in India have higher access to money and freedom to choose to go to market, there are less chances of having a stunted child as compared with women with less autonomy for such actions (Shroff *et al.* 2009). Gender inequality, poor empowerment of women and poor decision‐making powers adversely influence socio‐economic status and purchasing power, age of marriage and conception, choice of spacing between pregnancies, level of education, and experience of domestic violence, which in turn impact on women's status with serious implications on rate of childhood stunting. Studies from Pakistan, Bangladesh and India document the association of empowerment of women with food security, dietary diversity, appropriate infant feeding practices and improved growth outcomes (Bold *et al.* 2013). Gender inequality has been identified as an important factor that cannot be ignored in efforts to reduce stunting rate in South Asia (Smith & Haddad 2015). As discussed subsequently, gender inequalities such as indicated by early age of marriage and conception, poor rate of secondary education of women, low income and poor purchasing power of women play a crucial role in childhood stunting in South Asia.

### Early marriage, early conception and stunting in children

**Figure 3:**
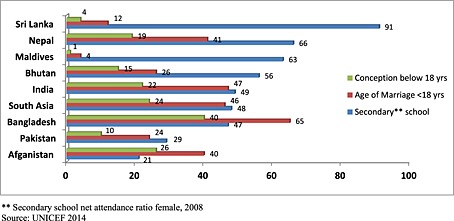
Status of women in South Asia: percentage with secondary education, low age of marriage and conception.

Data of South Asia countries reveal that marriage below 18 years and conception at a young age of below 20 years is common in six of the eight South Asia countries (Fig. 3) (UNICEF 2014b). Early marriage is often followed with early conception due to social pressure on newly married women to prove fertility. Early conception hinders optimum gain in height during adolescence – the second and last growth spurt of life. The capacity for catch‐up growth and attainment of final optimum adult size is further worsened in situations where these girls are reported to have been stunted at 3 years of age (Rao *et al.* 1998). The growth velocity of such stunted children is slow during adolescence with delayed growth spurt and elongated growth span. Early conception further hinders height gain.

Poor maternal height of women in turn increases chances of IUGR and LBW resulting in child anthropometric failure and stunting. It is reported that LBW and preterm delivery are twice as common in adolescent pregnancies than in adult pregnancies, while infants are 1.22 times at higher risk of stunting in situations where mothers are adolescent or below 18 years as compared with those over 18 years (Raj *et al.* 2010; Wu *et al.* 2012). Analysis of demographic data estimates that 8.6% of stunting cases in South Asia children could have been averted with elimination of teenage pregnancies and birth intervals of less than 24 months compared with a much lower impact of only 3.6% in the Middle East and North Africa (Fink *et al.* 2014).

### Education of mothers and stunting in children

Based on cross‐country studies, improvements in women's education have been reported to be responsible for almost 43% of the total reduction in underweight children between 1970 and 1995 (Bold *et al.* 2013). Investment in education and health of women is inextricably linked to improvement in nutrition of women and children (Nabarro *et al.* 2012). A study of 17 countries demonstrates a significant positive association between maternal education and nutritional status of children 3–23 months old (Cleland & Ginneken 1988; UNICEF 2009). The national demographic survey findings of India and Pakistan reveal that with increase in level of education of women, there is significant reduction in percentage of children with stunting as well as other important determinants of women's nutrition such as percentage with low age of marriage and age of first conception as well as per cent of mothers with low BMI and suffering domestic violence (NFHS‐3 2006; NNS 2011). Education empowers women, and secondary education could be considered a proxy indicator of improving decision‐making power of women. A study from Pakistan reveals that majority of children with signs of undernutrition had mothers who almost virtually had no schooling, and stunting rate dropped by almost 50% when mothers had secondary education compared with those with primary education (Liaqat *et al.* 2007). Reduction in child nutrition reported in Bangladesh has been associated with high rates of enrolment of girls in secondary education following introduction of subsidised education schemes for girls in the early 1990s (Headey *et al.* 2014). The following five overlapping pathways linking education and stunting have been proposed – transmission of information on health and nutrition, equipping mothers to acquire knowledge, increasing receptivity to modern medicine, increase in self‐confidence with positive impact on decision‐making and consulting health professionals, as well as enhancing social networking opportunities (Ruel *et al.* 2013).

### Domestic violence against women and child undernutrition

Domestic violence against women, an indicator of definite disempowerment, is common in South Asia with wife beating being acceptable by 52% of adult men and women in the region compared with only 20% in Central and Eastern Europe/Commonwealth of Independent States countries (UNICEF 2014b). Domestic violence resulting in psychological stress has been identified as a risk factor for preterm births and LBW (Hobel & Culhane 2003). The impact of violence is not limited to psychological and physical hazards. In South Asia, stress caused due to abuse during pregnancy is observed to be associated with both higher incidence of LBW and lower mean birth weight (Altarac & Strobino 2002). A study from Bangladesh indicates an association between experience or acceptance of physical domestic violence and child undernutrition and attributes this to lowering of self‐esteem and poor mental health, less control over household resources and access to usage of health services (Bhagowalia *et al.* 2012). The effect of domestic violence on nutrition and growth as well as operative pathways is understudied (Yount *et al.* 2011). India data demonstrate an association of multiple incidents of domestic violence with anaemia and underweight in women, which is hypothesised to be a result of increase in oxidative stress and metabolic levels (Ackerson & Subramanian 2008). A prospective study of rural Pakistan reveals that newborns of depressed mothers have higher level of growth retardation occurring in infancy (Rahman *et al.* 2004). Longitudinal studies in four countries, including Bangladesh, reports negative effects of domestic violence on birth weight and child's growth in the first 2 years of life with higher risk of stunting at 2 years of age as well as short stature at 7 years and adulthood (Yount *et al.* 2011). The possible factors leading to stunting is possibly through biological and behavioural pathways with adverse impact on fetal growth and pregnancy outcome as well as on self and child care behaviours (Yount *et al.* 2011; Charlette *et al.* 2012). Interestingly, the adverse impact of domestic violence on child nutrition and care weakens with increasing age of a child, possibly a reflection of reduced dependency on adults (Babu & Kar 2009; Charlette *et al.* 2012).

### Poverty, women's empowerment interventions and stunting

Association of poverty with stunting is evident from the significant difference noted in undernutrition rate in women and children in low wealth quintile compared with high wealth quintile. In India, the percentage of women with low BMI is 51.5% and stunted children is 59.9% in the lowest wealth index compared with 18.2% mothers with low BMI and 25.3% stunted children in the highest wealth index (NFHS‐3 2006). In Bhutan, children 0–23 months from the two lower wealth quintiles had 37% higher odds of being stunted as compared with children from two upper wealth quintiles (Aguayo *et al.* 2015). A recent analysis of Pakistan survey of 2011 reports that children are better nourished in situations where mothers are from wealthier households (Cesare *et al.* 2015).

Three types of social safety interventions in developing countries aim to empower women – cash transfer [conditional cash transfer (CCT) or unconditional cash transfer (UCT)], agriculture and microfinance programmes. These interventions aim at increasing purchasing power and thus empowering women to make better choices for self and family care with expected positive influence on nutritional status of women and children (IFPRI 2008). An analysis of cash transfer (CT) programmes reveal that CCT programme impacts on child anthropometry are mixed and little is known about the pathways through which nutrition outcome occurs (Bold *et al.* 2013). It is not clear whether the impact occurs due to conditionality influencing use of health or nutrition services and child care practices or due to other factors that impact improvement in quality of services and enhancement of knowledge which influence the desirable behavioural practices. Interestingly, the study also indicates that CCT interventions with non‐health conditionality such as savings or employment have negative impacts on nutritional status, while limited evidence from South Africa of UCT programme reports significant positive impacts on child nutrition. The findings of a recent comprehensive review of CT initiatives and child nutrition reveal a positive role of cash transfer programmes in enhancing resources for food, health and care (Groot *et al.* 2015). However, the evidence of impact of CT programmes on immediate determinants of child nutrition is reported to be mixed with reference to growth‐related outcomes among children. This study points out that ‘CT programmes with a larger transfer and a long duration, targeted at young children in low‐income households, with additional supply‐side interventions may have the greatest likelihood of success.’

Following the *Grameen Bank* Programme of Bangladesh, there has been a rapid increase in microfinance and rural livelihood programmes in South Asia with the key objective to empower the poor, particularly women. Rural livelihood programmes for female farmers in Gujarat and tribal belt of Rajasthan, India are reported to empower local communities, especially women with higher control over household finances, greater capacity to make decisions regarding health and education of children and higher autonomy. Information on impact of such interventions on improvements on nutritional status has not been systematically studied, but positive effects on behaviour are reported (Desai & Joshi 2012). Only in certain areas or regions with households living in stressed environment, microcredit programmes have been reported to have a positive effect on health and nutritional status of children (Stewart *et al.* 2010). It is hypothesised that impact of empowerment of women on nutrition outcome is possibly diluted by continued poor access to quality health services and sanitation (Transform Nutrition 2014). The evidence of microfinance programmes on women's empowerment measures as well as on nutritional status is limited and mixed with the pathway remaining unexplained. Evaluation designs are often weak or lack credibility (Bold *et al.* 2013).

### Women's agricultural activities, employment and child nutrition

In South Asia, unlike East Asia and developed economics, the largest share of women's employment is in agriculture (62.1%) followed by industry (17.3%) and services (16.6%) (UNCTAD 2011). Women's participation in the work force and its implications on childhood stunting has not been systematically studied. However, there is consensus on the following pathways through which targeted agricultural programmes for women influence nutrition – empowerment and decision‐making power, enhancement of social status, increase in control over resources including intrahousehold resource and time allocation for self and family care, which influence women's own health and nutritional status through impacting on dietary intake, energy expenditure and exposure to diseases (Ruel *et al.* 2013). Evidence indicates that agriculture interventions involve women are more likely to use the resulting increase in income for improving household security through positive influence in bargaining power of women within households and in making nutritionally appropriate choices with regard to household expenditure (Gillespie *et al.* 2012; Bold *et al.* 2013). On the other hand, a link between excess work during pregnancy and LBW and size is found to be more likely in case of children born to mothers engaged in agriculture work during pregnancy (Herforth 2012). A Pakistan study indicates that women employed in agriculture are three times more likely to be underweight compared with women who are not working and almost twice of those employed in non‐agriculture work (Balagamwala *et al.* 2015). It is also reported that in Pakistan, children of mothers working in agriculture are reported to have 52% stunted children compared with 42% in the case of non‐working mothers, 48% non‐agriculture workers and 44% in all mothers. Women's work in agriculture possibly increase resources available to the family but on the other hand may negatively impact on allocation of time or energy for child care and be a primary barrier in child feeding practices despite mothers having knowledge of appropriate feeding practices (Jones *et al.* 2012). However, a recent study from Nepal, using Women's Empowerment in Agriculture Index, reports that women's autonomy in production and women's work in agriculture influence diet diversity for children under 2 years old and reduce the incidence of stunting in children but not necessarily impacts women's nutritional status (Malapit *et al.* 2013).

Evidence of agriculture interventions on women's empowerment, time and workload is mixed. Impact of agriculture increases income as well as workload, but implications of these on child care are not well documented (Bold *et al.* 2013). No conclusive evidence on the effects of agriculture interventions in general or on nutritional status has been reported. However, a positive effect on dietary intake and nutritional status measures such as anthropometric indicators has been reported in most of the vegetable gardening interventions when combined with nutrition counselling component (Berti *et al.* 2004). A review of agricultural strategies, largely home gardens with or without animal production, reveals a positive impact on consumption of vitamin A‐rich fruits and vegetables, while the evidence of positive impact of targeted agriculture interventions on maternal and child nutrition is limited (Girard *et al.* 2012). In the last decade, Nepal has documented experience of combining home gardening project with intensive information‐education‐communication (IEC) activities, which resulted in higher consumption of special foods such as eggs, meat, milk, nuts and dried fruits during pregnancy (Jones *et al.* 2005). In the Helen Keller International Homestead Food Production (HKI‐HFP) project of Bangladesh and Nepal, agriculture and livestock support resulted in an increase in consumption of eggs in Bangladesh and of pulses and eggs in Nepal (Talukder *et al.* 2010; Girard *et al.* 2012). In these projects, a significant decline in anaemia is also observed. The evidence of such agriculture or home gardening strategies on anthropometric indicators is limited. It is not clear from the review of studies whether the impact on nutritional status is directly due to consumption of diversified foods produced or indirectly influenced through increase in purchasing power because agricultural strategies directed at women possibly influence income, livelihood and gender inequality (Girard *et al.* 2012). Agriculture interventions accompanied with nutrition education are likely to positively impact on nutrition outcomes (Bold *et al.* 2013). A need for further research in South Asia to measure and understand how agriculture affects nutrition through women's empowerment using the recently developed Women's Empowerment Agriculture Index is considered important.

## Looking towards the future in South Asia

Analysis of the situation in South Asia reveals that the coupling of nutrition‐sensitive interventions with the package of evidence‐based direct nutrition interventions in the first 1000 days is imperative (Bhutta *et al.* 2013). Women's empowerment and gender equality plays a central role in influencing women's health and nutrition. A recent report estimates that desirable improvement in gender inequality itself could contribute to 10 percentage points decline in stunting prevalence rate in children (Smith & Haddad 2015). There is an urgent need for intensifying interventions for the prevention of early age of marriage and conception, improving completion of secondary education by girls, improving access to diversified food and sanitation facilities, enhancing purchasing power and measures for reducing drudgery for water or fuel collection and directing efforts for elimination of domestic violence. Prevention of early marriage and early conception through investment in secondary level education combined with legislation enforcement efforts is crucial. Lessons could be derived from innovative incentivised secondary education programmes for girls in India and Bangladesh for scaling up such efforts in South Asia (Khanderker *et al.* 2003; MoWCD 2014). At the same time, it is also important that the emerging problem of overnutrition in women in South Asia is not ignored and opportunity of contacts with girls in school and with women at the workplace or any other community forum is actively used for imparting nutrition–health education.

Effective use of platforms of micro‐financing initiatives, livelihood programmes, cash transfer programme, agriculture interventions for reaching disadvantaged women with knowledge and information to facilitate them to make better choices for self, child and family care is vital. A good example of such a linkage is an innovative large‐scale experience from India of using microfinance programme for women as a platform for provision of one full hot cooked meal at a subsidised price to pregnant and lactating women along with intensifying nutrition–health education and ANC services with substantial improvement in weight gain during pregnancy and in reduction of LBW (Chava 2012).

Additionally, the CCT programme could also be designed with the objective to contribute to improve health and nutrition of women and reducing childhood stunting. CCT could be linked to conditions such as age of first conception >18 years, minimum of four antenatal visits, attending monthly weight‐monitoring sessions and gaining a minimum of 8 kg weight during pregnancy, compliance of at least 100 IFA tablets and opting for skilled birth delivery. Such conditions are also expected to contribute to increase in demand for better quality prenatal services for impacting on lowering incidence of LBW (Barber & Gertler 2010). Two CCT programmes of India [*Janani Surkasha Yojana (JSY)* and *Indira Gandhi Matritva Sahyog Yojna (IGMSY*) and the *Bangladesh Shombhob Conditional Cash Transfer*] are incentivized on conditions related to pregnancy, institutional delivery and /or child care and feeding practices (JSY 2005; IGMSY 2011; World Bank 2014) The experiences from these countries could provide lessons for introduction of such CCT schemes in other South Asia countries.

In South Asia, special care of women at pre‐conception stage is required to promote adequate weight gain and for elimination of anaemia. Vietnam and India experience of reaching newly married couples offers lessons towards formulating such a strategy (Khan *et al.* 2007; Vir 2013). Family planning interventions for newly married couples or marriage registration contacts are other opportunities that could be used not only for counselling on delaying conception but for improving weight and iron‐folic status of women prior to onset of pregnancy. Provision of balanced energy – protein supplements, with 25% energy contributed by protein – to ‘at risk’ women is essential (WHO 2014). Programme design for effective supplementation could be based on a critical study of India experience of Integrated Child Development Services programme, which reports a poor coverage, and the effective Bangladesh targeted food supplement initiative (NFHS‐3 2006; Ortolano *et al.* 2003). A locally suitable high dense nutrient food product with an appropriate distribution strategy could be developed to target pregnant women with low weight of 40–45 kg or height less than 145 cm or with low BMI < 18.5. Strengthening ANC services with higher priority to regular weight monitoring and counselling on appropriate weight gain during pregnancy is crucial. Irrespective of formal or informal sector, it is imperative that attention is also directed to support energy conservation in day to day work, and country policy is tightened towards provision of maternity protection benefits for ensuring an enabling environment for adequate rest and care during pregnancy and early childhood care.

For rapid reduction of stunting rates in children in South Asia, improving socio‐economic situation and decision‐making power of women must complement the ongoing efforts of improving coverage of the direct nutrition interventions. Political priority to formulate and implement an explicit policy on women's nutrition is essential towards reaching the 2010–2025 World Health Assembly goals of reducing the number of stunted children by 40%, LBW by 30% and anaemia in women of reproductive age group of women by 50% (WHO 2013).

## Source of funding

The author would like to acknowledge the financial support of UNICEF (ROSA).

## Conflicts of interest

The author declares that she has no conflicts of interest.

## Contributions

The manuscript drafted by the author, SCV, is based on the framework of the presentation made at the Regional Conference entitled ‘Stop Stunting: Improving Child Feeding, Women's Nutrition and Household Sanitation in South Asia’, organised by UNICEF, Regional Office of South Asia (ROSA) in November 2014. The ROSA team, comprising Dr Victor Aguayo and Dr Kajali Paintal contributed by making valuable comments in the overall focus of the presentation made at the conference.
